# Future COVID-19 Booster Vaccine Refusal in Healthcare Workers after a Massive Breakthrough Infection Wave, a Nationwide Survey-Based Study

**DOI:** 10.3390/vaccines11050987

**Published:** 2023-05-16

**Authors:** Fuying Gu, Huiying Lin, Zhenqiang Chen, Gareth Ambler, Xinyan Chen, Xiaoling Chen, Pincang Xia, Nan Liu, Houwei Du

**Affiliations:** 1 Department of Hepatobiliary Surgery, The Affiliated Hospital of Yangzhou University, Yangzhou University, Yangzhou 225012, China; gufuying781005@outlook.com; 2 Department of Neurology, Fujian Medical University Union Hospital, Fuzhou 350001, China; linhuiying202122@outlook.com (H.L.);; 3 Department of Rehabilitation, Fujian Medical University Union Hospital, Fuzhou 350001, China; 4 Statistical Science, University College London, London WC1E 6BT, UK; 5 Department of Infectious Disease, Fujian Medical University Union Hospital, Fuzhou 350001, China; 6 Fujian Center for Disease Control and Prevention, Fuzhou 350001, China

**Keywords:** coronavirus disease 2019, booster vaccine, healthcare workers, attitudes, survey

## Abstract

**Background:** An unprecedented coronavirus disease 2019 (COVID-19) wave occurred in China between December 2022 and January 2023, challenging the efficacy of the primary series of COVID-19 vaccines. The attitudes toward future COVID-19 booster vaccines (CBV) after the massive breakthrough infection among healthcare workers remain unknown. This study aimed to explore the prevalence and determinants of future CBV refusal after the unprecedented COVID-19 wave among healthcare workers. **Methods:** Between 9 and 19 February 2023, a cross-sectional nationwide online survey was conducted using a self-administered questionnaire vaccine among healthcare workers in China. Sociodemographics, profession, presence of chronic medical conditions, previous COVID-19 infection, attitudes towards future CBV, and reasons for future CBV refusal were collected. We estimated odds ratio [OR] with 95% confidence interval [CI] using a multivariable logistic regression model to explore the factors associated with future CBV refusal. **Results:** Among the 1618 participants who completed the survey, 1511 respondents with two or more doses of COVID-19 vaccines were analyzed. A total of 648 (41.8%) of respondents were unwilling to receive a future CBV. Multivariable logistic regression analysis revealed the association of CBV refusal with profession (vs. other staff, physician-adjusted OR 1.17, 95%CI 0.79–1.72, nurse-adjusted OR 1.88, 95%CI 1.24−2.85, *p* = 0.008), history of allergy (adjusted OR 1.72, 95%CI 1.05–2.83, *p* = 0.032), a lower self-perceived risk of future COVID-19 infection (*p* < 0.001), and a lower belief in CBV effectiveness (*p* = 0.014), safety (*p* < 0.001), and necessities for healthcare workers and the public (*p* < 0.001, respectively). **Conclusions:** Our findings highlight that a considerable proportion of healthcare workers were against a future booster dose after an unprecedented COVID-19 wave. Self-perception of future COVID-19 risk, and potential harm or doubtful efficacy of vaccines are the main determinants. Our findings may help public health authorities to establish future COVID-19 vaccination programs.

## 1. Introduction

Several types of vaccines have been introduced to prevent and control the coronavirus disease 2019 (COVID-19) pandemic caused by severe acute respiratory syndrome coronavirus (SARS-CoV-2) infection [1,2,3]. Generally, achieving adequate immunity requires a two-dose series of SARS-CoV-2 vaccines. However, accumulating evidence showed that the new Omicron virus variants could escape most therapeutic monoclonal antibodies and vaccine-elicited antibodies [4,5,6]. Substantial concerns have been raised regarding the duration of immunity after the primary two doses and potential breakthrough infection, suggesting a need for a COVID-19 booster vaccine (CBV) [7]. Some researchers argued that a fourth-dose booster might help maintain a high serum-neutralizing antibody level to protect against infection [8,9].

Since the introduction of the COVID-19 vaccine, nearly 3.5 billion doses of vaccines over different platforms have been administrated in China until 23 December 2022 [10]. Several previous observational studies showed that mild-to-moderate infections accounted for the majority of COVID-19 infection in China, 2022 [11,12,13]. Moreover, vaccination coverage has reached more than 85% in Mainland China, and each person has been inoculated with 2.2 doses on average [13]. Shortly after China moved away from its zero-COVID policy in late December 2022, an unprecedented COVID-19 wave spread in China. After reaching the peak (6.94 million) on 22 December 2022, it gradually decreased to 24,000 on 30 January 2023 [14]. Until 9 February 2023, there were 98,742 hospitalized COVID-19 patients, 7918 of whom experienced severe infection [15]. Healthcare workers are at a particularly high risk of SARS-CoV-2 infection, and their beliefs and attitudes toward CBV are extremely important for primary prevention strategies. Although some studies reported the attitudes toward CBV among healthcare workers [16,17], attitudes toward CBV may change over time because of the ever-changing public’s perception of COVID-19 risk and vaccination safety and efficacy. Data from Singaporean public primary care clinics showed that vaccine hesitancy among healthcare workers had improved from the first dose to CBV [18]. A cross-sectional study at the beginning of the fourth COVID-19 vaccination dose campaign in Israel showed that 53.9% of physicians were unwilling to be vaccinated compared to 83.3% of nurses and 69% of other healthcare professions, with the most frequent concerns regarding the vaccine being its efficacy, benefit, and necessity [19]. In the present study, we aimed to explore the prevalence and determinants of future CBV refusal at this particular time point.

## 2. Materials and Methods

### 2.1. Study Design and Participants

We conducted an online survey-based cross-sectional study between 9 and 19 February 2023, aiming to measure current attitudes towards future CBV among Mainland China healthcare workers. We also explored the factors that were associated with the future CBV refusal. This study was performed shortly after an unprecedented nationwide COVID-19 wave in Mainland China between December 2022 and January 2023 [14]. We utilized a self-administered questionnaire disseminated online through an app (Wen Juan Xing, Changsha Ranxing Information Technology Co., Ltd., Hunan, China) for data collection. Responses from participants were collected voluntarily and anonymously, with no personal data. We reported this study according to the Checklist for Reporting Results of Internet E-Surveys (CHERRIES) statement [20] and the American Association for Public Opinion Research (AAPOR) reporting guidelines for survey studies [21]. A minimum sample size of 1068 was calculated using an online sample size calculator (Rao soft, http://www.raosoft.com/samplesize.html (accessed on 31 January 2023)), with a 3% margin of error, 95% confidence interval, 50% response distribution, and targeted population size of 14 million [22], which was further inflated by 10% (*n* = 107), resulting in a final sample size of 1175. Invitations were sent to hospital-based and professional medical society-based networks, which then forwarded the invitations to their members, approximately 3000 individuals, to facilitate participation in the study. Agreeable participants may also invite other potential participants through their respective networks [16].

### 2.2. Questionnaire Design

Survey questions were developed by experts in primary care, disease control, infectious disease, and qualitative research, after referring to available systematic reviews [23,24,25]. The preliminary questionnaire focuses on previous COVID-19 infection and vaccination, self-perception of future COVID-19 risk, and attitudes towards a future CBV as well as its potential factors. The relevance, clarity, simplicity, and ambiguity of each question item were assessed using an ordinal four-point Likert scale (1 = not relevant, 2 = relevant but needs major revision, 3 = relevant but needs minor revision, 4 = very relevant) [24]. The content validity index was 0.88, calculated by the proportion of a score of 3 or 4 regarding each item content valid [26,27]. The improved questionnaire was administered in face-to-face interviews with 10 volunteer healthcare workers from different departments to determine whether the question items were well-understood. Five other healthcare workers completed the questionnaire twice at an interval of 10 days to test the reliability (the reliability coefficient was 0.898), with a median completion time of five minutes. The validity test of the questionnaire indicates good internal consistency (Cronbach’s alpha 0.809, KMO 0.782, Bartlett’s test of sphericity, *p* < 0.001). The final version of this questionnaire includes five domains (eMethods in Supplementary Materials). The first domain aims to collect sociodemographics (age, sex, marital status, profession, etc.) and health status variables. The second domain includes seven items regarding the previous COVID-19 infection, including onset symptoms (i.e., fever, cough) and disease severity (i.e., need for hospitalization or intensive care unit). The third section includes four items regarding COVID-19 vaccination history, including types of vaccines, number of doses, and adverse effects. The fourth domain aims to assess participants’ attitudes that could be associated with a future CBV refusal, addressing the perceived risk of future COVID-19 and the perceived benefits (preventing infection, preventing severe infection, etc.) and barriers (safety) of a CBV [28]. Responses for these questions were assessed using a three-point scale, with 1 = disagree, 2 = uncertain, and 3 = agree.

### 2.3. Outcomes

The first outcome was the refusal to receive a future CBV (yes/no) [29]. We explored the factors associated with the future CBV refusal. Secondary outcomes were the preference of the future CBV types and the drivers for the CBV acceptance and refusal. Additional outcomes included the prevalence and severity of previous COVID-19 infection.

### 2.4. Statistical Analysis

Continuous data were summarized as mean and standard variations [SD] or median with interquartile range (IQR) and categorical variables with frequencies with percentage, where appropriate. Missing data of weight (*n* = 16) and height (*n* = 22) were imputed using a conditional specification method while taking into account the joint distribution of age and sex. There were no missing data for other variables. We compared between-groups differences in continuous variables using the t-test or the Mann–Whitney test, and categorical variables using the Chi-square test or Fisher’s exact test as appropriate. A multivariable logistic regression model was used to evaluate the adjusted odds ratio (OR) of the CBV refusal for each variable with a *p* value < 0.2 in the univariable analysis. We conducted two sensitivity analyses, by limiting the sample to those who had previous COVID-19 or excluding those who had received the fourth dose. All analyses were conducted using SPSS Statistics Version 25.0 (IBM, Chicago, IL, USA), and significance was set at α = 0.05.

## 3. Results

The questionnaire was completed by 1618 participants with a median completion time of 296 (IQR 227–399) seconds. A total of 1285 (78.4%) respondents had received three to four doses by the time of responding to this survey, 266 (16.4%) received two doses, 27 (1.5%) received one dose, and 40 (2.5%) were unvaccinated. Table S1 shows the demographics and characteristics among participants with different doses. In total, 1551 participants (median age of 38 [IQR 31–46], female 64.1%) who received two or more doses were included in the final analysis. Of them, 1416 (91.3%) participants had previous COVID-19 infection confirmed via nucleic acid tests. Sociodemographics and characteristics among those with and without previous COVID-19 are shown in Table 1. The most commonly reported symptom was cough (1273 [89.9]%), followed by fever (1217 [85.9%]), and fatigue (1214 [85.7%], Figure S1). COVID-19 pneumonia was reported in 67 (4.7%) participants, no pneumonia in 1003 (70.8%), and uncertainty in 346 (24.4%). Only 17 (1.2%) infections were asymptomatic. The most reported administered vaccine was inactivated vaccine (1465 [94.5%]), followed by viral vector vaccine (21 [1.4%]), protein submit vaccine (13 [0.8%]), and mRNA vaccines (1 [0.06%]). A total of 51 (3.3%) participants could not recall the vaccine type administered (Figure S2). Regarding the post-vaccination adverse effects, 1218 (78.5%) reported none, 294 (19.0%) reported mild-to-moderate local adverse effects, 36 (2.3%) reported mild-to-moderate systemic adverse effects, and only 3 (0.2%) reported severe side effects (Figure S3). Adverse (or side) effects after previous COVID-19 vaccine types are shown in Table S2.

### 3.1. Acceptance and Refusal of Future CBV

A total of 648 (41.8%) participants were unwilling to receive a future booster dose. Table 2 summarizes the differences in the sociodemographics and characteristics among acceptance and refusal groups. Participants in the refusal and acceptance groups differed in the proportions of female (446 [68.8%] vs. 548 [60.7%], *p* = 0.001), profession (*p* < 0.001), frequency of physical activity (*p* = 0.007), history of allergy (70 [10.8%] vs. 71 [7.9%], *p* = 0.047), post-vaccination adverse effects (*p* = 0.002), having COVID-19 cohabitants (*p* = 0.022), and living alone (53 [8.2%] vs. 115 [12.7%], *p* = 0.004). Regarding the self-perception of the future COVID-19 risk, the participants in the refusal group were less likely to agree “There will be a future COVID-19 wave in 2023” (241 [37.2%] vs. 408 [45.2%]), and “I will get a future COVID-19 in 2023” (147 [16.3%] vs. 158 [24.4%], Table 3). Regarding the attitudes towards the future CBV, participants in the refusal group were less likely to agree “CBV could terminate the COVID-19 pandemic” (21 [3.2%] vs. 100 [11.1%]), “CBV could prevent future COVID-19” (55 [8.5%] vs. 327 [36.2%]), “CBV could prevent severe COVID-19” (179 [27.6%] vs. 581 [64.3%]), “CBV is necessary to healthcare workers” (97 [15.0%] vs. 647 [71.7%]), “CBV is necessary to the public” (39 [6.0%] vs. 494 [54.7%]), and “CBV is safe” (135 [20.8%] vs. 639 [70.8%], Table 3).

### 3.2. Determinants for Future CBV

Multivariable regression analysis showed that CBV refusal was significantly associated with profession (vs. other staff, physician-adjusted OR 1.17, 95%CI 0.79–1.72, nurse-adjusted OR 1.88, 95%CI 1.24–2.85, overall *p* = 0.008), history of allergy (adjusted OR 1.72, 95%CI 1.05–2.83, *p* = 0.032), a lower self-perceived risk of future COVID-19 risk (vs. agree “I will get a future COVID-19 in 2023”, disagree-adjusted OR 2.16, 95%CI 1.37–3.38), and a lower belief in CBV effectiveness (vs. agree “CBV could prevent future COVID-19”, disagree-adjusted OR 2.09, 95%CI 1.27–3.44), safety (vs. agree “I thank CBV is safe”, uncertain-adjusted OR 3.66, 95%CI 2.65–5.12; disagree-adjusted 10.95, 95%CI 2.51–47.87, overall *p* < 0.001), and necessities for the healthcare workers as well as the public (overall *p* < 0.001, respectively, Table 4). Sensitivity analysis limited to those with previous COVID-19 (n = 1416) or excluding those who had received the fourth dose (n = 1425) did not alter the main findings (Tables S3 and S4).

### 3.3. Preference of Future CBV Types, and Drivers for Future CBV Refusal

Among the 903 respondents who were willing to receive the future CBV, inactivated vaccines account for 494 (54.7%), followed by inhaled vaccines 119 (13.2%), mRNA vaccines 91 (10.0%), viral vector vaccines 24 (2.7%), and bivalent vaccines 12(1.3%). A total of 164 (18.0%) respondents did not care about the vaccine types. Regarding the drives for the vaccine acceptance, 560 believed “CBV is effective”, 488 believed “CBV is safe”, and 373 chose the option “others”. Among the 648 participants who were against the future CBV, the top three reasons for the future CBV refusal included concerns about efficacy (365 [56.3%]), necessity (161 [24.8%]), and safety (101 [15.6%], Table S5).

## 4. Discussion

This is the first study, to our knowledge, to explore the prevalence and associated factors of future CBV refusal after an unprecedented COVID-19 wave in fully vaccinated healthcare workers in China. Our study has several important findings. First, nearly half of the respondents were against a future CBV. Second, CBV refusal was significantly associated with profession (nurse), history of allergy, a lower self-perceived risk of future infection, and a lower belief in CBV effectiveness, safety, and necessities for healthcare workers and the public.

Compared to findings of available literatures [30,31], the prevalence of previous COVID-19 infection was higher in our participants. Thus, such a high infection prevalence in our participants may indicate massive breakthrough infections among fully vaccinated healthcare workers in Mainland China. One possible explanation is that the Omicron variants can escape antibody neutralization from the serum of both previously infected and vaccinated individuals [32,33]. Previous studies showed that the protective effect of the third dose only lasted for a short time against Omicron variants [34,35]. Moreover, data from Israel showed that the fourth-dose mRNA vaccine had short-term benefits in reducing the odds of severe COVID-19, hospitalization, and mortality in old individuals who had received the third dose more than 4 months previously [36,37]. Additionally, among triple-vaccinated healthcare workers who had a previous COVID-19 infection, the boosting effect of Omicron is minimal [38]. The aforementioned findings may suggest the potential need for a future booster dose in high-risk populations.

Nearly half of our respondents indicated a refusal to receive a future CBV. In contrast to our data, several previous literatures reported high vaccination acceptance in healthcare workers [39,40]. A previous study in China showed that 87% of respondents were willing to receive a CBV [40]. A review paper showed that the prevalence of hesitancy for COVID-19 vaccination varied from 4.3% to 72% among healthcare workers worldwide [41]. Possible explanations for the disparities might include policy and socio-cultural differences and different time points [39,42,43]. Due to the issues of methodology (for example, imbalanced response options) and self-selection of respondents, the prevalence of refusal is likely to be overestimated. 

Our multivariable regression analyses showed that respondents who refused to receive a future CBV more likely disagreed with its necessity and safety. Such a high proportion (91%) of our vaccinated participants reported previous COVID-19 infection; thus, the experience of potential poor protective effect of vaccination could be a factor influencing the willingness to receive a future CBV. Concerns about effectiveness (32%) and possible long-term adverse effects (31%) accounted for the top reasons of the CBV refusal among Indian and Saudi Arabian healthcare workers [29]. Moreover, a cross-sectional study in China showed that post-vaccination adverse effects were associated with a lower willingness to receive a booster dose (OR = 0.37, 95%CI: 0.23–0.59) [40]. More than half (54.7%) of our respondents preferred inactivated vaccines as a future CBV shot. This might be explained by the low prevalence (0.2%) of severe adverse effects reported following inactivated vaccination in our participants. All of the aforementioned findings suggest that addressing concerns about vaccination efficacy and safety should be further incorporated into communication campaigns and healthcare systems to improve CBV acceptance and uptake intent.

Our regression analyses showed that nurses had higher odds of a future CBV refusal compared to other staff. Our results are comparable to findings of other studies that showed vaccination intention varies with profession [44,45,46,47]. Data from Singapore showed that hesitancy in nurses (15 [7.4%]) was higher than for staff in medical (0%) and applied health (5 [3.5%]) [45] professions. A cross-sectional study among Israel healthcare workers showed that 83.3% of nurses were against the fourth dose [19]. Possible explanations might include more concerns about vaccine effectiveness and safety, and less knowledge about the vaccine among nurses [48], since nurses were less likely to have a higher education background among the different professions of our participants (Table S6). Our findings suggest that immediate attention is needed on the strategies to promote the uptake of CBV for nurses.

Our data showed an association of CBV refusal with a lower perceived risk of future COVID-19 and a lower belief in CBV effectiveness. Our findings were comparable to those of previous studies. A previous survey showed that participants who agreed that the CBV could control severe COVID-19 (OR 3.18, 95%CI 2.00–5.05) and symptomatic SARS-CoV-2 infection (OR 2.77, 95%CI 1.81–4.23) were more likely to accept a booster dose [44]. Moreover, the top three reasons for COVID-19 vaccine acceptance among healthcare workers in Singapore were “To protect themselves from COVID-19”, “To protect their family and friends”, and “Due to high risk of acquiring COVID-19 because of their jobs” [45]. A scoping review showed that participants who had direct contact with COVID-19 patients or a higher self-perceived infection risk were predictors for a higher vaccine acceptance among healthcare workers [41].

### Strengths and Limitations

Our study was included a high proportion of vaccinated participants who also reported previous COVID-19 infection. Moreover, the harvested sample from the anonymous online survey has an optimal size and decent representativeness to reflect several essential characteristics of the target population (i.e., sex, marital status, and professional groups). We acknowledge limitations. First, this cross-sectional survey with a non-random sample strategy inevitably leads to selection bias and self-reporting bias. For example, our participants were relatively young, partly because younger healthcare workers may be more willing to participate in the online survey. Additionally, those with strong views towards COVID-19 vaccination are more likely to respond to a survey. Thus, the sample may not be fully representative. Moreover, selection bias might also be introduced since respondents from the same network may share similar opinions and viewpoints. However, we did not have information on potential clusters and were thus unable to adjust for this in our analysis. Second, data regarding the proportion of respondents agreeing or refusing to the future CBV among the different society networks were unavailable. Third, this study used a self-administered questionnaire, and the results were not externally validated. Fourth, our findings may not be generalized to other populations since healthcare workers are more aware of the risk-benefit profile of COVID-19 vaccines. The generalization and external validity should be investigated in further studies. Lastly, the response rate could not be calculated in this online survey [49].

## 5. Conclusions

This study identified several determinants for the future boost dose refusal among healthcare workers in Mainland China, including profession, a lower self-perceived risk of future COVID-19 infection, and a lower belief in CBV effectiveness, safety, and necessity. Healthcare workers might have a role model effect on the public; thus, a comprehensive understanding their motivations for CBV refusal might be informative to future vaccination campaign strategies. The present study may help public health authorities to establish future COVID-19 vaccination programs.

## Figures and Tables

**Table 1:** Characteristics of participants with and without previous COVID-19.

	Total (n = 1551)	Without COVID-19 (n = 135)	With COVID-19 (n = 1416)	*p*-Value
**Sociodemographics**				
Age, y, median (IQR)	38 (31–46)	38 (31–49)	38 (31–45)	0.369
Female sex, n (%)	994 (64.1)	75 (55.6)	919 (64.9)	0.031
Marital status				0.024
Divorced or widowed, n (%)	20 (1.3)	5 (3.7)	15 (1.1)	
Unmarried, n (%)	321 (20.7)	31 (23.0)	290 (20.5)	
Married, n (%)	1210 (78.0)	99 (73.3)	1111 (78.5)	
Live with children, n (%)	901 (58.1)	67 (49.6)	834 (58.9)	0.037
Live with old, n (%)	747 (48.2)	54 (40.0)	693 (48.9)	0.047
Live alone, n (%)	168 (10.8)	21 (15.6)	147 (10.4)	0.065
Live with dependents, n (%)	52 (3.4)	3 (2.2)	49 (3.5)	0.445
Education				0.964
Below bachelor ’s degree, n (%)	195 (12.6)	16 (11.9)	179 (12.6)	
Bachelor’s degree, n (%)	851 (54.9)	75 (55.6)	776 (54.8)	
Postgraduate’s degree, n (%)	505 (32.6)	44 (32.6)	461 (32.6)	
Types of hospital				0.882
Community hospital, n (%)	88 (5.7)	8 (5.9)	80 (5.6)	
Non-designated hospital, n (%)	973 (62.7)	82 (60.7)	891 (62.9)	
Designated hospital, n (%)	490 (31.6)	45 (33.3)	445 (31.4)	
Profession				0.206
Physician, n (%)	734 (47.3)	70 (51.9)	664 (46.9)	
Nurse, n (%)	475 (30.6)	33 (24.4)	442 (31.2)	
Other staff, n(%)	342 (22.1)	32 (23.7)	310 (21.9)	
Years of practice, y median [IQR]	14 (7–24)	14 (6–28)	14 (7–23)	0.413
**Health status**				
BMI, kg/m^2^, mean (SD)	22.71 (3.03)	23.16 (3.25)	22.67 (3.01)	0.069
Frequency of physical activity				0.310
Never or rare, n (%)	611 (39.4)	45 (33.3)	566 (39.4)	
Seldom, n (%)	710 (45.8)	67 (49.6)	643 (45.4)	
Often to frequent, n (%)	230 (14.8)	207 (14.6)	23 (17.0)	
Current smoker, n (%)	77 (5.0)	14 (10.4)	63 (4.4)	0.001
Regular alcohol user, n (%)	43 (2.8)	4 (3.0)	39 (2.8)	0.921
History of allergy, n (%)	141 (9.1)	12 (8.9)	129 (9.1)	0.932
History of chronic disease *, n (%)	576 (37.1)	56 (41.5)	520 (36.7)	0.274
**Previous COVID-19 infection**				
Cohabitation infection				<0.001
Infected, n (%)	1375 (88.7)	74 (54.9)	1301 (91.8)	
Unknown, n (%)	60 (3.9)	5 (3.7)	55 (3.9)	
Not infected, n (%)	116 (7.5)	56 (41.5)	60 (4.2)	
Family infection				<0.001
Infected, n (%)	1404 (90.6)	87 (64.4)	1317 (93.0)	
Unknown, n (%)	40 (2.6)	4 (3.0)	36 (2.5)	
Not infected, n (%)	107 (6.9)	44 (32.6)	63 (4.4)	
Post-vaccination adverse effects				0.387
None, n (%)	1218 (78.5)	104 (77.0)	1114 (78.7)	
Local, n (%)	294 (19.0)	28 (20.7)	266 (18.8)	
Systemic or severe, n (%)	39 (2.5)	3 (2.2)	36 (2.5)	

*, History of choronic disease = Any of the following: hypertension, hyperlipidemia, diabetes, chronic heart disease, immune disease, and tumor. Abbreviation: BMI = body mass index; COVID-19 = coronavirus disease 2019; IQR = interquartile range; SD = standard deviation.

**Table 2:** Characteristics among booster vaccine acceptance and refusal groups.

	Total (n = 1511)	Acceptance (n = 903)	Refusal (n = 648)	*p* Value
**Sociodemographics**				
Age, y, median (IQR)	38 (31–46)	38 (31–45)	39 (32–46)	0.199
Female sex, n (%)	994 (64.1)	548 (60.7)	446 (68.8)	0.001
Marital status				0.505
Divorced or widowed, n (%)	20 (1.3)	14 (1.6)	6 (0.9)	
Unmarried, n (%)	321 (20.7)	190 (21.0)	131 (20.2)	
Married, n (%)	1210 (78.0)	699 (77.4)	511 (78.9)	
Live with children, n (%)	901 (58.1)	517 (57.3)	384 (59.3)	0.430
Live with old, n (%)	747 (48.2)	450 (49.8)	296 (45.8)	0.120
Live alone, n (%)	168 (10.8)	115 (12.7)	53 (8.2)	0.004
Live with the dependents, n (%)	52 (3.4)	29 (3.2)	23 (3.5)	0.715
Education				0.474
Below bachelor ’s degree, n (%)	195 (12.6)	115 (12.7)	80 (12.3)	
Bachelor’s degree, n (%)	851 (54.9)	484 (53.6)	367 (56.6)	
Postgraduate’s degree, n (%)	505(32.6)	304 (33.7)	01 (31.0)	
Types of hospital				0.170
Community hospital, n (%)	88 (5.7)	55 (6.1)	33 (5.1)	
Non-designated hospital, n (%)	973 (62.7)	579 (64.1)	394 (60.8)	
Designated hospital, n (%)	490 (31.6)	269 (29.8)	221 (34.1)	
Profession				<0.001
Physician, n (%)	734 (47.3)	451 (49.9)	283 (43.7)	
Nurse, n (%)	475 (30.6)	235 (26.0)	240 (37.0)	
Other staff, n (%)	342 (22.1)	217 (24.1)	125 (19.3)	
Years of practice, y median (IQR)	14 (7–24)	14 (6–24)	14 (7–23)	0.475
Had direct COVID-19 patients contact, n (%)	1311 (84.5)	756 (83.7)	555 (85.6)	0.301
**Health status**				
BMI, kg/m^2^, mean [SD]	22.71 (3.03)	22.78 (3.01)	22.62 (3.06)	0.250
Frequency of physical activity Rare or never, n (%) Sometimes, n (%) Often to frequent, n (%)				0.007
	611 (39.4) 710 (45.8)	328 (36.3) 442 (48.9)	283 (43.7) 268 (41.4)	
	230 (14.8)	133 (14.7)	97 (15.0)	
Current smoker, n (%)	77 (5.0)	51 (5.6)	26 (4.0)	0.338
Often drinker, n (%)	43 (2.8)	21 (2.3)	22 (3.4)	0.127
History of allergy, n (%)	141 (9.1)	71 (7.9)	70 (10.8)	0.047
Chronic disease *, n (%)	576 (37.1)	343 (38.0)	233 (36.0)	0.415
**Previous COVID-19 history**				
Previous COVID-19 infection	1416 (91.3)	812 (89.9)	604 (93.2)	0.024
Previous COVID-19 pneumonia	68 (4.4)	38 (4.2)	30 (4.6)	0.923
Cohabitant COVID-19 infection				0.022
Infected, n (%)	1375 (88.7)	784 (86.8)	591 (91.3)	
Uncertain, n (%)	60 (3.9)	43 (4.8)	17 (2.6)	
Not infected, n (%)	116 (7.5)	76 (8.4)	40 (6.2)	
Family COVID-19 infection				0.233
Infected, n (%)	1404 (90.6)	816 (90.3)	587 (90.6)	
Unknown, n (%)	40 (2.6)	23 (2.5)	17 (2.6)	
Not infected, n (%)	107 (6.9)	63 (7.0)	44 (6.8)	
**Previous vaccination**				
Post-vaccination adverse effects				0.004
None, n (%)	1218 (78.5)	737 (81.6)	481 (74.2)	
Local, n (%)	294 (19.0)	149 (16.5)	145 (22.4)	
Systemic or severe, n (%)	39 (2.5)	17 (1.9)	22 (3.4)	

* Chronic disease = Any of the following: hypertension, hyperlipidemia, diabetes, chronic heart disease, immune disease, and tumor. Abbreviation: BMI = body mass index; COVID-19 = coronavirus disease 2019; IQR = interquartile range; SD = standard deviation.

**Table 3:** Self-perception of future COVID-19 and a booster vaccine among booster vaccine acceptance and refusal groups.

	Total	Acceptance	Refusal	*p*-Value
	(n = 1511)	(n = 903)	(n = 648)	
**Perception of future COVID-19**				
There will be a future wave in 2023				0.003
Agree, (%)	649 (41.8)	408 (45.2)	241 (37.2)	
Uncertain, (%)	633 (40.8)	357 (39.5)	276 (42.6)	
Disagree, n (%)	269 (17.3)	138 (15.3)	131 (20.2)	
COVID-19 will be similar to flu				0.088
Agree, n (%)	653 (42.1)	400 (44.3)	253 (39.0)	
Uncertain, n (%)	558 (36.0)	307 (34.0)	251 (38.7)	
Disagree, n (%)	340 (21.9)	196 (21.7)	144 (22.2)	
I will get a future COVID-19				<0.001
Agree, n (%)	576 (37.1)	345 (38.2)	231 (35.6)	
Uncertain, n (%)	670 (43.2)	411 (45.5)	259 (40.0)	
Disagree, n (%)	305 (19.7)	147 (16.3)	158 (24.4)	
My future COVID-19 will need hospitalization				0.216
Agree, n (%)	21 (1.4)	9 (1.0)	12 (1.9)	
Uncertain, n (%)	271 (17.5)	151 (16.7)	120 (18.5)	
Disagree, n (%)	1259 (81.2)	743 (82.3)	516 (79.6)	
CBV could terminate COVID-19				<0.001
Agree, n (%)	121 (7.8)	100 (11.1)	21 (3.2)	
Uncertain, n (%)	590 (38.0)	369 (40.9)	221 (34.1)	
Disagree, n (%)	840 (54.2)	434 (48.1)	406 (62.7)	
CBV could prevent future COVID-19				<0.001
Agree, n (%)	382 (24.6)	327 (36.2)	55 (8.5)	
Uncertain, n (%)	686 (44.2)	387 (42.9)	299 (46.1)	
Disagree, n (%)	483 (31.1)	189 (20.9)	294 (45.4)	
CBV could prevent future severe COVID-19				<0.001
Agree, n (%)	760 (49.0)	581 (64.3)	179 (27.6)	
Uncertain, n (%)	581 (37.5)	280 (31.0)	301 (46.5)	
Disagree, n (%)	210 (13.5)	42 (4.7)	168 (25.9)	
**Self-perception of CBV**				
CBV is necessary for healthcare workers				<0.001
Agree, n (%)	744 (48.0)	647 (71.7)	97 (15.0)	
Uncertain, n (%)	465 (30.0)	190 (21.0)	275 (42.4)	
Disagree, n (%)	342 (22.1)	66 (7.3)	276 (42.6)	
CBV is necessary for public				<0.001
Agree, n (%)	533 (34.3)	494 (54.7)	39 (6.0)	
Uncertain, n (%)	701 (45.2)	312 (34.6)	389 (60.0)	
Disagree, n (%)	317 (20.4)	97 (10.7)	220 (34.0)	
I think CBV is safe				<0.001
Agree, n (%)	774 (49.9)	639 (70.8)	135 (20.8)	
Uncertain, n (%)	738 (47.6)	261 (28.9)	477 (73.6)	
Disagree, n (%)	39 (2.5)	3 (0.3)	36 (5.6)	

Abbreviation: COVID-19 = coronavirus disease 2019; CBV = COVID-19 booster dose vaccine.

**Table 4:** Predictors for the future booster dose refusal.

	Unadjusted OR [95% CI]	*p*-Value	Adjusted OR [95% CI]	*p*-Value
Age	0.99 [0.98–1.00]	0.187	1.00 [0.98–1.02]	0.989
Female sex	1.43 [1.16–1.77]	0.001	1.14 [0.77–1.70]	0.520
Live alone	1.64 [1.16–2.31]	0.005	0.72 [0.43–1.23]	0.233
Live with old	1.17 [0.96–1.44]	0.120	0.87 [0.65–1.17]	0.372
Profession		<0.001		0.008
Other staff	Ref		Ref	
Physician	1.09 [0.84–1.42]		1.17 [0.79–1.72]	
Nurse	1.77 [1.33–2.36]		1.88 [1.24–2.85]	
Types of hospital		0.170		0.776
Community	Ref		Ref	
Non-designated	1.13 [0.72–1.78]		0.79 [0.42–1.50]	
Designated	1.37 [0.86–2.18]		0.81 [0.42–1.57]	
Frequency of physical activity		0.007		0.899
Never or rare	Ref		Ref	
Sometimes	0.70 [0.56–0.88]		0.93 [0.68–1.27]	
Often to frequent	0.85 [0.62–1.15]		0.99 [0.63–1.55]	
Drinker		0.129		0.908
Never or rare	Ref		Ref	
Occasional	0.84 [0.68–1.04]		0.94 [0.66–1.34]	
Regular	1.39 [0.75–2.56]		1.10 [0.43–2.83]	
History of allergy	1.42 [1.00–2.01]	0.048	1.72 [1.05–2.83]	0.032
Previous COVID-19	1.54 [1.06–2.24]	0.024	1.60 [0.91–2.81]	0.100
Cohabitating with COVID-19 infection		0.022		0.184
Infected	Ref		Ref	
Uncertain	0.75 [0.38–1.48]		0.54 [0.21–1.38]	
Not infected	1.43 [0.96–2.13]		1.12 [0.61–2.04]	
Post-vaccination adverse effects		0.002		0.995
None	Ref		Ref	
Local	1.49 [1.15–1.93]		1.02 [0.70–1.47]	
Systemic or severe	1.98 [1.04–3.77]		1.01 [0.39–2.59]	
There will be a future wave in 2023		0.003		0.665
Agree	Ref		Ref	
Uncertain	1.31 [1.05–1.64]		0.87 [0.60–1.27]	
Disagree	1.61 [1.21–2.14]		1.06 [0.66–1.68]	
Future COVID-19 will be similar to flu		0.088		0.777
Agree	Ref		Ref	
Uncertain	1.16 [0.89–1.52]		1.10 [0.72–1.68]	
Disagree	1.29 [1.03–1.63]		0.96 [0.64–1.42]	
I will get a future COVID-19 in 2023		<0.001		<0.001
Agree	Ref		Ref	
Uncertain	0.94 [0.75–1.18]		0.93 [0.66–1.309]	
Disagree	1.61 [1.21–2.12]		2.16 [1.37–3.38]	
CBV could terminate COVID-19 pandemic		<0.001		0.096
Agree	Ref		Ref	
Uncertain	0.64 [0.52–0.79]		0.66 [0.45–0.96]	
Disagree	0.22 [0.14–0.37]		0.84 [0.40–1.76]	
CBV could prevent future COVID-19		<0.001		0.014
Agree	Ref		Ref	
Uncertain	4.59 [3.33–6.34]		1.50 [0.91–2.47]	
Disagree	9.25 [6.58–12.98]		2.09 [1.27–3.44]	
CBV could prevent severe COVID-19		<0.001		0.088
Agree	Ref		Ref	
Uncertain	3.49 [2.76–4.41]		0.92 [0.62–1.37]	
Disagree	12.98 [8.90–18.94]		1.73 [1.00–2.99]	
I think CBV is safe		<0.001		<0.001
Agree	Ref		Ref	
Uncertain	8.65 [6.81–10.99]		3.66 [2.65–5.12]	
Disagree	56.80 [17.24–187.14]		10.95 [2.51–47.87]	
CBV is necessary for healthcare workers		<0.001		<0.001
Agree	Ref		Ref	
Uncertain	9.65 [7.28–12.80]		3.55 [2.41–5.24]	
Disagree	27.89 [19.79–39.31]		6.36 [4.10–9.85]	
CBV is necessary for the public		<0.001		<0.001
Agree	Ref		Ref	
Uncertain	15.79 [11.31–22.60]		4.21 [2.69–6.59]	
Disagree	28.73 [19.18–43.04]		7.30 [4.42–12.04]	

Abbreviation: CI = confidence interval; COVID-19 = coronavirus disease 2019; CBV = COVID-19 booster dose vaccine; OR = odds ratio; IQR = interquartile range.

## Data Availability

The datasets used and/or analyzed during the current study are available from the corresponding author on reasonable request.

## Supplementary Materials

The following supporting information can be downloaded at: https://www.mdpi.com/article/10.3390/vaccines11050987/s1. eMethods: Questionnaire. Figure S1: Self-reported COVID-19 symptoms. Figure S2: Administered COVID-19 vaccines. Figure S3: Post-COVID-19 vaccination adverse effects. Table S1: Demographics among participants with different doses. Table S2. Adverse (or side) effects after previous COVID-19 vaccine types. Table S3: Sensitivity analysis limited to those with previous COVID-19. Table S4: Sensitivity analysis excluding those who had received the fourth dose. Table S5: Reasons for future COVID-19 booster vaccine refusal. Table S6: Demographics and characteristics among participants with different profession.

## Abbreviations

BMI: body mass index; CBV: COVID-19 booster vaccine; CI: confidence interval; COVID-19: coronavirus disease 2019; IQR: interquartile range; OR: odds ratio; SARS-CoV-2: severe acute respiratory syndrome coronavirus SD: standard variations.
