# Supplementary material for: Future COVID-19 Booster Vaccine Refusal in Healthcare Workers after a Massive Breakthrough Infection Wave, a Nationwide Survey-Based Study
Source: Vaccines (Basel). 2023 May 16;11(5):987. doi: 10.3390/vaccines11050987 (PMC10222960; doi:10.3390/vaccines11050987)
Supplement: Supplementary file 1 [file vaccines-11-00987-s001.zip › vaccines-2327357-supplementary.pdf]

Title: Future COVID-19 Booster Vaccine Refusal in Healthcare Workers after a Massive Breakthrough Infection Wave, a Nationwide Survey-Based Study

Online supplemental material

1. eMethods: Questionnaire
2. Supplemental Figures.
Figure S1: Self-reported COVID-19 symptoms
Figure S2: Administered COVID-19 vaccines
Figure S3: Post-COVID-19 vaccination adverse effects
3. Supplemental Tables
Table S1: Demographics among participants with different doses
Table S2, Adverse (or side) effects after previous COVID-19 vaccine types
Table S3: Sensitivity analysis limited to those with previous COVID-19
Table S4: Sensitivity analysis excluding those who had received the fourth dose
Table S5. Reasons for future COVID-19 booster vaccine refusal
Table S6: Demographics and characteristics among participants with different profession

eMethods: Questionnaire

Introduction:

Dear healthcare workers, you are invited to participate in a survey regarding your attitudes towards a future COVID-19 booster vaccine. This questionnaire consists 42 items, and will be completed within about 5 minutes. We guarantee this survey is voluntary and anonymous, with no personal data. Many thanks for your contribution.

Section 1: Sociodemographics and health status (17 items)

1. Age ___ (years, continuous variable)
2. Gender Male Female
3. Height ___ (cm, continuous variable)
4. Weight ___ (kg, continuous variable)
5. Martial status Married Unmarried Divorced or widow
6. Education background Below bachelor's degree Bachelor's degree Postgraduate's degree
7. Profession Physician Nurse Other staff
8. Your hospital type Community hospital Non-designated hospital Designated hospital

Note: Designated hospital = hospital designated for COVID-19 patients

9. Working department Respiratory Intensive care unit Emergency Others
10. Years of practice___ (years, continuous variable)
11. Had direct COVID-19 patients contact? Yes No
12. Who do you live with? (Multiple choice) Children Old people Dependent Other people Live alone
13. Do you smoke? Current smoker Ex-smoker Non-smoker
14. Do you drink? Regular Occasional Never or rare
15. What's your frequency of physical activity Often Seldom Rare or never
16. Do you have a history of allergy? Yes No
17. Do you have chronic disease (Multiple choice): Hypertension; Hyperlipidemia; Diabetes; Chronic heart disease; Asthma; Chronic obstructive pulmonary disease; Lung nodule; Chronic liver disease; Chronic renal disease; Tumor; Insomnia; Anxiety; Depression; Additional medical history___ (Please describe)

Section 2. Self-report of previous SARS-CoV-2 infection (7 items)

1. Do your family members have a COVID-19 infection? Yes No
2. Were your family members hospitalized due to COVID-19? Yes No
3. What is your COVID-19 infection status?
   Infected or recovered
   Re-infected
   Not infected

If you have a previous COVID-19 infection, please respond to items 4 - 7

4. Severity of your previous infection
   Not severe
   Severe but not hospitalized
   Severe and hospitalized
5. What COVID-19 symptoms did you suffer? (Multiple choice): Fever; Cough; Expectoration; Catarrh; Chest distress; Dyspnea; Chestpain; Myalgia; Fatigue; Digestive symptoms; Headache; Insomnia; Hyposmia; Hypogeusia; Anxiety; Depression

6. Do you have pneumonia caused by previous COVID-19 infection   Yes   No
7. How long do you recover? ___ (Days, continuous variable)

Section 3. Self-report of previous COVID-19 vaccination (4 items)

1. How many doses did you receive?
   Four doses   Three doses   Two doses   One dose   None
2. What type of vaccine have you received?
   Inactivated   protein subunit vaccines   viral vector vaccines   mRNA vaccines
3. Time from your recent vaccination ___ (days, continuous variable)
4. Do you experience post-COVID-19 vaccination adverse effects?
   Local but not severe   Systemic but not severe   Severe   None

Mild to moderate (not severe) local adverse effects include injection site pain, redness, and swelling, without the need for emergency medical care or hospitalization. Mild to moderate (not severe) systemic adverse reactions refer to fever, fatigue, chills, etc, without the need for emergency medical care or hospitalization. Severe side effects refer to those (i.e., shock, severe allergy, thrombosis) that need emergency medical care or hospitalization.

Section 4. Self-perception of future COVID-19 risk (4 items)

1. There will be a future COVID-19 wave in 2023   Agree   Uncertain   Disagree
2. Future COVID-19 will be similar to flu   Agree   Uncertain   Disagree
3 I will get a future COVID-19 in 2023   Agree   Uncertain   Disagree
4. If I get a future COVID-19 infection, it will be severe   Agree   Uncertain   Disagree

Section 5. Attitudes towards a future COVID-19 booster vaccinates (CBV) (10 items)

1. CBV could terminate COVID-19 pandemic   Agree   Uncertain   Disagree
2. CBV could prevent future COVID-19 infection   Agree   Uncertain   Disagree
3. CBV could prevent future severe COVID-19   Agree   Uncertain   Disagree
4. CBV is necessary for healthcare workers   Agree   Uncertain   Disagree
5. CBV is necessary for the public   Agree   Uncertain   Disagree
6. I think CBV is safe   Agree   Uncertain   Disagree
7. Are you willing to receive a future CBV?   Yes   No
8. Reasons for your willingness of receiving a booster vaccination (Multiple choice)
   CBV is effective
   CBV is safe
   Other reasons
9. Type of booster vaccine you are willing to receive (Multiple choice)
   Inactivate COVID-19 vaccines
   Inhalation recombination COVID-19 vaccines
   mRNA vaccines
   viral vector vaccines
   Bivalent COVID-19 vaccine
   Monovalent COVID-19 vaccine
10. Reason for your unwillingness to receive a booster vaccine (Multiple choice)
   Concerns about effectiveness
   Concerns about safety
   Concerns about economical factors
   I think it is not necessary

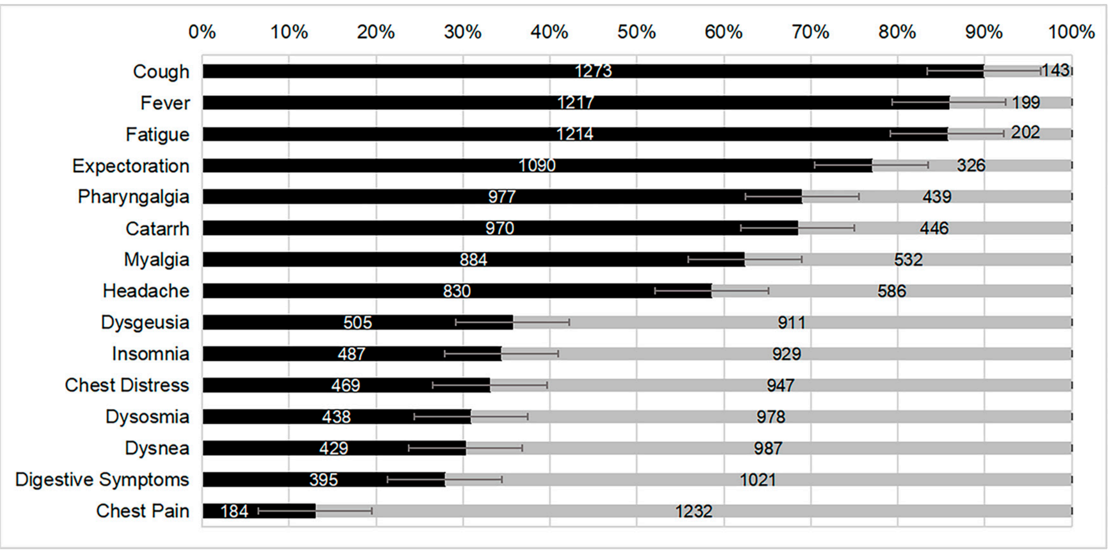

Figure S1: Self-reported COVID-19 symptoms

Abbreviation: COVID-19 = coronavirus disease 2019;

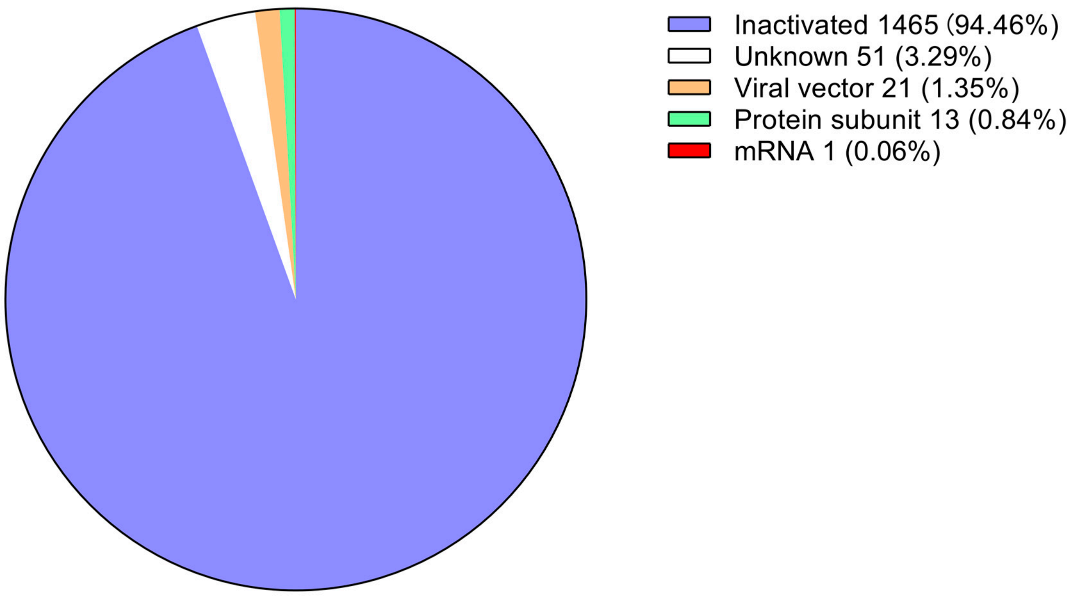

Figure S2: Administered COVID-19 vaccines

Abbreviation: COVID-19 = coronavirus disease 2019;

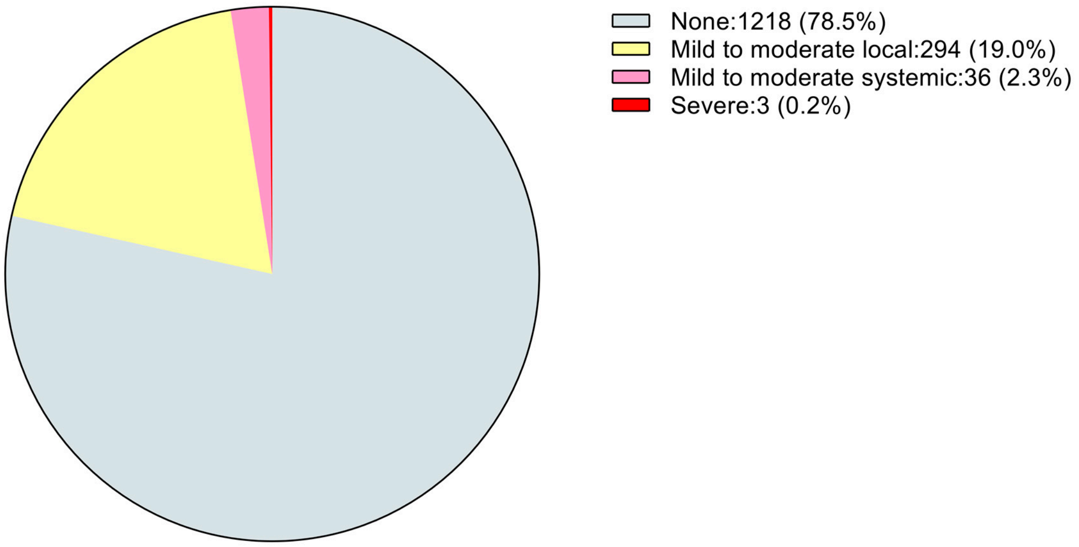

Figure S3: Post COVID-19 vaccination adverse effects

Abbreviation: COVID-19 = coronavirus disease 2019;

Table S1. Demographics among participants with different doses (n = 1618)

	Total (n =1618)	Four doses (n = 126)	Three doses (n = 1159)	Two doses (n = 226)	One dose (n = 27)	None (n = 40)
**Sociodemographics**						
Age, y, median (IQR)	38 (31 - 46)	43 (36 - 48)	38 (31 - 46)	36 (30 - 43)	35 (31 -45)	38 (33 - 44)
Female sex, n (%)	1046 (64.6)	47 (37.3)	756 (65.2)	191 (71.8)	21 (77.8)	31 (77.5)
Martial status						
Divorced, n (%)	25 (1.5)	1 (0.8)	17 (1.5)	2 (0.8)	3 (11.1)	2 (5.0)
Unmarried, n (%)	332 (20.5)	14 (11.1)	245 (21.1)	62 (23.3)	6 (22.2)	5 (12.5)
Married, n (%)	1261 (77.9)	111 (88.1)	897 (77.4)	202 (75.9)	18 (66.7)	33 (82.5)
Live with children, n (%)	942 (58.2)	84 (66.7)	659 (56.9)	158 (59.4)	14 (51.9)	27 (67.5)
Live with old, n (%)	778 (48.1)	64 (50.8)	564 (48.7)	119 (44.7)	12 (44.4)	19 (47.5)
Live alone, n (%)	177 (10.9)	10 (7.9)	125 (10.8)	33 (12.4)	5 (18.5)	4 (10.0)
Live with dependent, n (%)	55 (3.4)	3 (2.4)	32 (2.8)	17 (6.4)	1 (3.7)	2 (5.0)
Education						
Below bachelor ’s degree, n (%)	198 (12.2)	6 (4.8)	161 (13.9)	28 (10.5)	2 (7.4)	1 (2.5)
Bachelor’s degree, n (%)	877 (54.2)	68 (54.0)	645 (55.7)	138 (51.9)	10 (37.0)	16 (40.0)
Postgraduate’s degree, n (%)	543 (33.6)	52 (41.3)	353 (30.5)	100 (37.6)	15 (55.6)	23 (57.5)
Type of hospital						
Community hospital, n (%)	90 (5.6)	11 (8.7)	68 (5.9)	9 (3.4)	1 (3.7)	1 (2.5)
Non-designated hospital, n (%)	1024 (63.3)	73 (57.9)	726 (62.6)	174 (65.4)	21 (77.8)	30 (75.0)
Designated hospital, n (%)	504 (31.1)	42 (33.3)	365 (31.5)	83 (31.2)	5 (18.5)	9 (22.5)
Profession						
Physician, n (%)	775 (47.9)	92 (73.0)	518 (44.7)	124 (46.6)	16 (59.3)	25 (62.5)
Nurse, n (%)	491 (30.3)	13 (10.3)	380 (32.8)	82 (30.8)	6 (22.2)	10 (25.0)

Other staff, n (%)	352 (21.8)	21 (16.7)	261 (22.5)	60 (22.6)	5 (18.5)	5 (12.5)
Department						
Respiratory, n (%)	42 (2.6)	6 (4.8)	31 (2.7)	2 (0.8)	2 (7.4)	1 (2.5)
Emergent, n (%)	57 (3.5)	5 (4.0)	45 (3.9)	6 (2.3)	1 (3.7)	0 (0.0)
Intensive care unit, n (%)	64 (4.0)	5 (4.0)	48 (4.1)	8 (3.0)	0 (0.0)	3 (7.5)
Others, n (%)	1455 (89.9)	110 (87.3)	1035 (89.3)	250 (94.0)	24 (88.9)	36 (90.0)
Had direct COVID-19 patients contact, n (%)	1373 (84.9)	112 (88.9)	983 (84.8)	216 (81.2)	25 (92.6))	37 (92.5)
Yeasr of practice, y, median (IQR)	14 [7 - 23]	18 [11 - 24]	14 [7 -24]	11 [5 -20]	11 [4 - 20]	12 [8 - 20]
**Health status**						
BMI, kg/m^2, mean (SD)	22.71 (3.03)	23.62 (2.90)	22.65 (2.98)	22.55 (3.25)	22.12 (3.28)	22.63 (2.35)
Current smoker, n (%)	79 (4.9)	12 (9.5)	59 (5.1)	6 (2.3)	1 (3.7)	1 (2.5)
Drinker						
Never or rare, n (%)	1016 (62.8)	65 (51.6)	723 (62.4)	179 (67.3)	19 (70.4)	30 (75)
Occasional, n (%)	559 (34.5)	53 (42.1)	405 (34.9)	83 (31.2)	8 (29.6)	10 (25.0)
Regular	43 (2.7)	8 (6.3)	31 (2.7)	4 (1.5)	0	0
Frequency of physical activity						
Rare or never, n (%)	633 (39.1)	47 (37.3)	444 (38.3)	120 (45.1)	9 (33.3)	13 (32.5)
Sometimes, n (%)	744 (46.0)	56 (44.4)	537 (46.3)	117 (44.0)	13 (48.1)	21 (52.5)
Ofter to frequent, n (%)	241 (14.9)	23 (13.8)	178 (15.4)	29 (10.9)	5 (18.5)	6 (15.0)
History of allergy, n (%)	152 (9.4)	7 (5.6)	102 (8.8)	32 (12.0)	4 (14.8)	7 (17.5)
History of chronic disease*, n (%)	595 (36.8)	56 (44.4)	424 (36.6)	96 (36.1)	9 (33.3)	10 (25.0)
**Previous COVID-19 history**						
Previous COVID-19 infection, n (%)	1476 (91.7)	86 (68.3)	1083 (93.4)	247 (92.9)	25 (92.6)	35 (87.5)
COVID - 19 pneumonia, n (%)	72 (15.7)	4 (13.3)	49 (14.9)	15 (19.2)	2 (25.0)	2 (13.3)
Cohabitation COVID-19 infection						
Infected, n (%)	1430 (99.4)	102 (80.9)	1044 (90.1)	229 (86.1)	20 (74.0)	35 (87.5)

Unknown, n (%)	64 (4.0)	4 (3,2)	43 (3.7)	13 (4.9)	2 (7.4)	2 (5.0)
Not infected, n (%)	124 (7.7)	20 (15.9)	72 (6.2)	24 (9.0)	5 (18.5)	3 (7.5)
Family COVID-19 infection						
Infected, n (%)	1463 (90.4)	106 (84.1)	1062 (91.6)	236 (88.8)	23 (85.2)	36 (90.0)
Unknown, n (%)	42 (2.6)	4 (3.2)	29 (2.5)	7 (2.6)	1 (3.7)	1 (2.5)
Not infected, n (%)	113 (7.0)	16 (12.7)	68 (5.9)	23 (8.6)	3 (11.1)	3 (7.5)
Post-vaccination adverse effects						
None, n (%)	1267 (78.3)	100 (79.4)	924 (79.7)	194 (72.9)	12 (44.4)	37 (92.5)
Local, n (%)	299 (18.5)	25 (19.8)	209 (18.0)	60 (22.6)	4 (14.8)	1 (2.5)
Systemic or severe, n (%)	52 (3.2)	1 (0.8)	26 (2.2)	12 (4.5)	11 (40.7)	2 (5.0)

*Chronic disease = Any of the following: hypertension, hyperlipidemia, diabetes, chronic heart disease, immune disease, and tumor

Abbreviation: BMI = Body mass index; COVID-19 = coronavirus disease 2019; IQR = interquartile range; SD = standard deviation

Table S2, Adverse (or side) effects after previous COVID-19 vaccine types

	Inactivated	Viral vector	Protein subunit	mRNA	Unknown
None	1161 (79.2%)	8 (38.1%)	9 (69.2%)	0	40 (78.4%)
Local	267 (18.2%)	11 (52.4%)	4 (30.8%)	1 (100%)	11 (21.6%)
Systemic	34 (2.3%)	2 (9.5%)	0	0	0
Severe	3 (0.2%)	0	0	0	0

Table S3. Predictors for booster dose refusal in those with previous COVID-19 (n = 1416)

	Unadjuted OR [95% CI]	P-value	Adjuted OR [95% CI]	P - value
Age	0.99 [0.98 - 1.00]	0.257	1.00 [0.98 - 1.02]	0.932
Female sex	1.34 [1.07 - 1.68]	0.010	0.99 [0.65 - 1.50]	0.946
Live alone	0.54 [0.37 - 0.78]	0.001	0.60 [0.34 -1.07]	0.084
Live with old	0.85 [0.68 - 1.04]	0.117	0.88 [0.65 - 1.20]	0.421
Profession		<0.001		0.011
Other staff	Ref		Ref	
Physician	1.02 [0.77 - 1.35]		1.00 [0.67 - 1.50]	
Nurse	1.70 [1.26 - 2.78]		1.75 [1.14 -2.70]	
Frequency of physical activity		0.033		0.984
Never or rare	Ref		Ref	
Sometimes	0.74 [0.59 - 0.93]		0.99 [0.71 - 1.36]	
Often to frequent	0.85 [0.61 - 1.17]		0.96 [0.60 - 1.54]	
Drinker		0.173		0.861
Never	Ref		Ref	
Seldom	0.84 [0.67 - 1.05]		0.91 [0.63 - 1.31]	
Often	1.34 [0.71 - 2.55]		1.05 [0.39 - 2.86]	
History of allergy	1.51 [1.05 - 2.17]	0.026	1.87 [1.11 - 3.14]	0.019
Cohabitant infection		0.032		0.193
Not infected	Ref		Ref	
Uncertain	0.51 [0.23 - 1.14]		0.60 [0.20 - 1.74]	
Infected	1.16 [0.68 - 1.96]		1.27 [0.61 - 2.65]	
Post-vaccination adverse effects		0.004		0.902
None	Ref		Ref	
Local	1.52 [1.16 -1.99]		1.05 [0.72 - 1.55]	
Syetemic or severe	1.65 [0.85 - 3.21]		0.84 [0.31 - 2.25]	
There will be a future wave in 2023		0.003		0.978
Agree	Ref		Ref	
Uncertain	1.37 [1.09 - 1.73]		0.97 [0.65 - 1.43]	
Disagree	1.60 [1.18 - 2.16]		0.96 [0.59 - 1.56]	
Future COVID-19 will be similar to flu		0.131		0.732
Agree	Ref		Ref	
Uncertain	1.10 [0.83- 1.45]		1.18 [0.76 - 1.84]	
Disagree	1.28 [1.00 - 1.62]		1.15 [0.76 - 1.75]	
"I will get a future COVID-19 in 2023"		<0.001		<0.001
Agree	Ref		Ref	
Uncertain	0.98 [0.78 - 1.24]		0.92 [0.65 - 1.31]	
Disagree	1.74 [1.29 - 2.33]		2.24 [1.40 - 3.60]	
CBV could terminate		<0.001		0.113

COVID-19 pandemic				
Disagree	Ref		Ref	
Uncertain	4.42 [2.67 - 7.32]		1.18 [0.55 - 2.54]	
Agree	2.83 [1.69 - 4.73]		0.78 [0.36 - 1.70	
CBV could prevent future COVID-19		<0.001		0.009
Agree	Ref		Ref	
Uncertain	4.62 [3.30 -6.46]		1.53 [0.91 -2.57]	
Disagree	9.39 [6.59 - 13.38]		2.24 [1.33 - 3.75]	
CBV could prevent future severe COVID-19		<0.001		0.146
Agree	Ref		Ref	
Uncertain	3.40 [2.67 - 4.33]		0.96 [0.64 - 1.45]	
Disagree	12.11 [8.15 - 17.98]		1.71 [0.97 - 3.04]	
I think CBV is safe		<0.001		<0.001
Agree	Ref		Ref	
Uncertain	8.62 [6.72 - 11.07]		3.73 [2.66 - 5.24]	
Disagree	44.36 [13.30 - 147.93]		10.34 [2.17 - 49.23]	
CBV is necessary for healthcare workers		<0.001		<0.001
Agree	Ref		Ref	
Uncertain	8.94 [6.68 - 11.96]		3.08 [2.05 - 4.61]	
Disagree	28.30 [19.69 - 40.68]		6.34 [4.00 - 10.06]	
CBV is necessary for the public		<0.001		<0.001
Agree	Ref		Ref	
Uncertain	15.85 [10.91 - 23.03]		4.45 [2.66 - 5.24]	
Disagree	29.81 [19.50 - 45.56]		7.62 [4.51 - 12.88]	

Abbreviation: CI = confidence interval; COVID-19 = coronavirus disease 2019; CBV = COVID-19 booster dose vaccine; OR = odds ratio;

Table S4. Predictors for booster dose refusal in those with two or three doses (n = 1425)

	Unadjuted OR [95% CI]	P-value	Adjuted OR [95% CI]	P - value
Age	1.00 [0.99 - 1.01]	0.569	1.01 [0.99 - 1.03]	0.262
Female sex	0.77 [0.61 - 0.96]	0.020	1.11 [0.73 - 1.69]	0.615
Live alone	0.57 [0.40 - 0.81]	0.002	0.75 [0.43 - 1.29]	0.292
Live with old	0.86 [0.70 - 1.06]	0.165	0.92 [0.68 - 1.25]	0.596
Profession		<0.001		0.014
Other staff	Ref		Ref	
Physician	1.14 [0.86 - 1.50]		1.25 [0.84 - 1.87]	
Nurse	1.73 [1.29 - 2.31]		1.86 [1.22 -2.83]	
Frequency of physical activity		0.004		0.568
Never or rare	Ref		Ref	
Sometimes	0.68 [0.54 - 0.85]		0.85 [0.61 - 1.18]	
Often to frequent	0.83 [0.60 - 1.14]		0.83 [0.52 -1.34]	
Drinker		0.076		0.915
Never or rare	Ref		Ref	
Seldom	0.87 [0.69 - 1.08]		0.96 [0.66 - 1.38]	
Regular	1.84 [0.92 - 3.67]		1.17 [0.40 - 3.43]	
History of allergy	1.35 [0.94 - 1.92]	0.103	1.62 [0.97 - 2.70]	0.065
Previous COVID-19			1.23 [0.65 - 2.34]	0.528
Cohabitant infection		0.082		0.147
Infected	Ref		Ref	
Uncertain	0.64 [0.32 - 1.28]		0.54 [0.20 - 1.44]	
Not infected	1.19 [0.78 - 1.82]		1.19 [0.63 -2.27]	
Post-vaccination adverse effects		0.001		0.970
None	Ref		Ref	
Local	1.55 [1.18 - 2.02]		1.04 [0.71 - 1.53]	
Systemic or severe	1.93 [1.00 - 3.72]		0.94 [0.36 -2.49]	
There will be a future wave in 2023		0.003		0.726
Agree	Ref		Ref	
Uncertain	1.28 [1.02 - 1.62]		0.92 [0.62 -1.36]	
Disagree	1.67 [1.24 - 2.26]		1.12 [0.69 -1.82]	
Future COVID-19 will be similar to flu		0.198		0.867
Agree	Ref		Ref	
Uncertain	1.17 [0.88 - 1.54]		1.11 [0.72 - 1.73]	
Disagree	1.24 [0.98- 1.57]		1.02 [0.67 -1.54]	
I will get a future COIVD-19 in 2023		<0.001		<0.001
Agree	Ref		Ref	
Uncertain	0.97 [0.77 - 1.23]		1.02 [0.72 - 1.45]	

Disagree	1.73 [1.29 - 2.32]		2.47 [1.53 - 3.99]	
CBV could terminate COVID-19 pandemic		<0.001		0.119
Disagree	Ref		Ref	
Uncertain	4.59 [2.80 - 7.53]		1.09 [0.51 - 2.31]	
Agree	2.86 [1.73 - 4.73]		0.72 [0.33 - 1.56]	
CBV could prevent future COVID-19		<0.001		0.006
Agree	Ref		Ref	
Uncertain	4.78 [3.43 -6.65]		1.61 [0.96 -2.69]	
Disagree	10.16 [7.15 - 14.45]		2.34 [1.40 - 3.94]	
CBV could prevent future severe COVID-19		<0.001		0.049
Agree	Ref		Ref	
Uncertain	3.53 [2.77 - 4.50]		0.96 [0.63 - 1.45]	
Disagree	13.61 [9.13 - 20.31]		1.98 [1.11 - 3.54]	
I think CBV is safe		<0.001		<0.001
Agree	Ref		Ref	
Uncertain	8.25 [6.45 - 10.56]		3.45 [2.46 - 4.84]	
Disagree	51.23 [15.54 - 168.94]		9.55 [2.12 - 42.95]	
CBV is necessary for healthcare workers		<0.001		<0.001
Agree	Ref		Ref	
Uncertain	9.55 [7.15 - 12.77]		3.35 [2.24 - 5.00]	
Disagree	30.59 [21.20 - 44.15]		6.32 [3.98 - 10.03]	
CBV is necessary for the public		<0.001		<0.001
Agree	Ref		Ref	
Uncertain	16.55 [11.43 - 23.94]		4.77 [3.01 - 7.56]	
Disagree	30.44 [19.99 - 46.36]		7.74 [4.59 - 13.04]	

Abbreviation: CI = confidence interval; COVID-19 = coronavirus disease 2019; CBV = COVID-19 booster dose vaccine; OR = odds ratio;

Table S5. Reasons for future COVID-19 booster vaccine refusal

Items	Number	Percentage
Concerns about safety	101	15.6%
Concernsabout efficacy	365	56.3%
Concerns about necessity	161	24.8%
Concerns about economic factor	4	0.6%
Concerns about other factors (for example, time consuming)	65	10.0%

Abbreviation: CI = confidence interval; COVID-19 = coronavirus disease 2019;

Table S6. Characteristics of participants with different profession (n = 1511)

	Nurse (n = 475)	Physician (n = 734)	Other staff (n = 342)	P-value
**Sociodemographics**				
Age, y, median (IQR)	37 (30 -45)	40 (33 - 46)	37 (29 - 46)	0.001
Female sex, n (%)	469 (98.7)	294 (40.1)	231 (67.5)	<0.001
Martial status				<0.001
Unmarried, n (%)	83 (17.5)	140 (19.1)	98 (28.7)	
Divorced or widowed, n (%)	3 (0.6)	12 (1.6)	5 (1.5)	
Married, n (%)	389 (81.9)	582 (79.3)	239 (69.9)	
Live with children, n (%)	281 (59.2)	441 (60.1)	179 (52.3)	0.048
Live with old, n (%)	237 (49.9)	363 (49.5)	147 (43.0)	0.094
Live alone, n (%)	50 (10.5)	68 (9.3)	50 (14.6)	0.030
Live with dependents, n (%)	23 (4.8)	21 (2.9)	8 (2.3)	0.087
Education				<0.001
Below bachelor ’s degree, n (%)	126 (26.5)	12 (1.6)	57 (16.7)	
Bachelor’s degree, n (%)	348 (73.3)	287 (39.1)	216 (63.2)	
Postgraduate’s degree, n (%)	1 (0.2)	435 (59.3)	69 (20.2)	
Types of hospital				<0.001
Community hospital, n (%)	20 (4.2)	35 (4.8)	33 (9.6)	
Non-designated hospital, n (%)	268 (56.4)	489 (66.6)	216 (63.2)	
Designated hospital, n (%)	187 (39.4)	210 (28.6)	93 (27.2)	
Department				0.002
Respiratory, n (%)	16 (3.4)	21 (2.9)	2 (0.6)	
Emergency n (%)	19 (4.0)	31 (4.2)	6 (1.8)	
Intensive care unit, n (%)	26 (5.5)	30 (4.1)	5 (1.5)	
Others, n (%)	414 (87.2)	652 (88.8)	329 (96.2)	
Years of practice, y median IQR)	15 (8 -26)	14 (7 - 22)	12 (5 -23)	0.001
Direct contact COVID-19 patients, n (%)	427 (89.9)	674 (91.8)	210 (61.4)	<0.001
**Health status**				
BMI, kg/m^2, mean (SD)	22.0 (2.9)	23.0 (3.3)	22.6 (3.8)	<0.001
Frequency of physical activity				0.005
Rare or never, n (%)	220 (46.3)	264 (36.0)	127 (37.1)	
Sometimes, n (%)	198 (41.7)	353 (48.1)	159 (46.5)	
Often to frequent, n (%)	57 (12.0)	117 (15.9)	56 (16.4)	
Current smoker, n (%)	0 (0.0)	49 (6.7)	28 (8.2)	<0.001
Regular drinker, n (%)	0 (0.0)	32 (4.4)	11 (3.2)	<0.001
History of allergy, n (%)	62 (13.1)	56 (7.6)	23 (6.7)	0.001
History of chronic disease*, n (%)	158 (33.3)	302 (41.1)	116 (33.9)	0.008
**Previous COVID-19 history**				

Previous COVID-19 infection	442 (93.1)	664 (90.5)	310 (90.6)	0.263
COVID-19 Pneumonia, n (%)	29 (6.1)	27 (3.7)	12 (3.5)	0.026
Cohabitation COVID-19 infection				0.006
Infection, n(%)	425 (89.5)	652 (88.8)	298 (87.1)	
Unknown, n (%)	16 (3.4)	29 (4.0)	15 (4.4)	
Not infected, n (%)	34 (7.2)	53 (7.2)	29 (8.5)	
Family member infection				<0.001
Infected, n(%)	434 (91.4)	670 (91.2)	300 (87.8)	
Unknown, n (%)	11 (2.3)	16 (2.2)	13 (3.8)	
Not infected, n (%)	30 (6.3)	48 (6.5)	29 (8.5)	
Previous vaccination				
Vaccine doses, n (%)				<0.001
Two to three doses	462 (97.3)	642 (87.5)	321 (93.9)	
Four doses	13 (2.7)	92 (12.5)	21 (6.1)	
Post-vaccination adverse effects				0.291
None, n (%)	383 (80.6)	579 (78.9)	256 (74.9)	
Local, n (%)	81 (17.1)	138 (18.8)	75 (21.9)	
Systemic or severe, n (%)	11 (2.3)	17 (2.3)	11 (3.2)	
**Perception of future COVID-19**				
There will be a future wave in 2023				<0.001
Agree, n (%)	161 (33.9)	348 (47.4)	140 (40.9)	
Uncertain, n (%)	258 (54.3)	232 (31.6)	143 (41.8)	
Disagree, n (%)	56 (11.8)	154 (21.0)	59 (17.3)	
Future COVID -19 will be similar to flu				<0.001
Agree, n (%)	197 (41.5)	331 (45.1)	125 (36.5)	
Uncertain, n (%)	201 (42.3)	215 (29.3)	142 (41.5)	
Disagree, n (%)	77 (16.2)	188 (25.6)	75 (21.9)	
I will get a future COVID-19 in 2023				<0.001
Agree, n (%)	141 (29.7)	310 (42.2)	125 (36.5)	
Uncertain, n (%)	232 (48.8)	300 (40.9)	138 (40.4)	
Disagree, n (%)	102 (21.5)	124 (16.9)	79 (23.1)	
My future COVID-19 will need hospitalization				0.129
Agree, n (%)	8 (1.7)	8 (1.1)	5 (1.5)	
Uncertain, n (%)	98 (20.6)	118 (16.1)	55 (16.1)	
Disagree, n (%)	369 (77.7)	608 (82.9)	282 (82.4)	
CBV would terminate COVID-19 pandemic				<0.001
Agree, n (%)	35 (7.4)	52 (7.1)	34 (9.9)	
Uncertain, n (%)	231 (48.6)	220 (30.0)	139 (40.6)	
Disagree, n (%)	209 (44.0)	462 (62.9)	169 (49.4)	
CBV would prevent future infection				<0.001
Agree, n (%)	93 (19.6)	196 (26.7)	93 (27.2)	
Uncertain, n (%)	266 (56.0)	272 (37.1)	148 (43.3)	
Disagree, n (%)	116 (24.4)	266 (36.2)	101 (29.5)	

CBV would prevente severe infection				<0.001
Agree, n (%)	196 (41.3)	393 (53.5)	171 (50.0)	
Uncertain, n (%)	220 (46.3)	230 (31.3)	131 (38.3)	
Disagree, n (%)	59 (12.4)	111 (15.1)	40 (11.7)	
Self-perception of CBV				
CBV is necessary for healthcare workers				<0.001
Agree, n (%)	204 (42.9)	364 (49.6)	176 (51.5)	
Uncertain, n (%)	182 (38.3)	180 (24.5)	103 (30.1)	
Disagree, n (%)	89 (18.7)	190 (25.9)	63 (18.4)	
CBV is necessary for the public				<0.001
Agree, n (%)	146 (30.7)	266 (36.2)	121 (35.4)	
Uncertain, n (%)	263 (55.4)	285 (38.8)	153 (44.7)	
Disagree, n (%)	66 (13.9)	183 (24.9)	68 (19.9)	
I think CBV is safe				<0.001
Agree, n (%)	180 (37.9)	420 (57.3)	174 (50.9)	
Uncertain, n (%)	287 (60.4)	296 (40.3)	155 (45.3)	
Disagree, n (%)	8 (1.7)	18 (2.5)	13 (3.8)	
Acceptance of a future CBV	235 (49.5)	451 (61.4)	217 (63.5)	<0.001
Concerns about effectiveness	142 (29.9)	287 (39.1)	131 (38.3)	0.003
Concerns about safety	116 (24.4)	244 (33.2)	128 (37.4)	<0.001

*Chronic disease = Any of the following: hypertension, hyperlipidemia, diabetes, chronic heart disease, immune disease, and tumor

Abbreviation: BMI = body mass index; COVID-19 = coronavirus disease 2019; CBV = COVID-19 booster dose vaccine; IQR = interquartile range; SD = standard deviation；
